# Atomic Diffusion-Induced Polarization and Superconductivity in Topological Insulator-Based Heterostructures

**DOI:** 10.1021/acsnano.3c08601

**Published:** 2023-12-21

**Authors:** Xian-Kui Wei, Abdur Rehman Jalil, Philipp Rüßmann, Yoichi Ando, Detlev Grützmacher, Stefan Blügel, Joachim Mayer

**Affiliations:** † Ernst Ruska-Centre for Microscopy and Spectroscopy with Electrons, Forschungszentrum Jülich GmbH, 52425 Jülich, Germany; ‡ Peter Grünberg Institute and JARA-FIT, Forschungszentrum Jülich GmbH, 52425 Jülich, Germany; § Institute for Theoretical Physics and Astrophysics, University of Würzburg, 97074 Würzburg, Germany; ∥ Peter Grünberg Institute and Institute for Advanced Simulation, Forschungszentrum Jülich GmbH and JARA, 52425 Jülich, Germany; ⊥ Physics Institute II, University of Cologne, Zülpicher Straße 77, 50937 Köln, Germany; ▽ Central Facility for Electron Microscopy, RWTH Aachen University, Ahornstraße 55, 52074 Aachen, Germany

**Keywords:** topological insulator, superconductivity, polarization, atomic diffusion and intercalation, scanning transmission electron microscopy

## Abstract

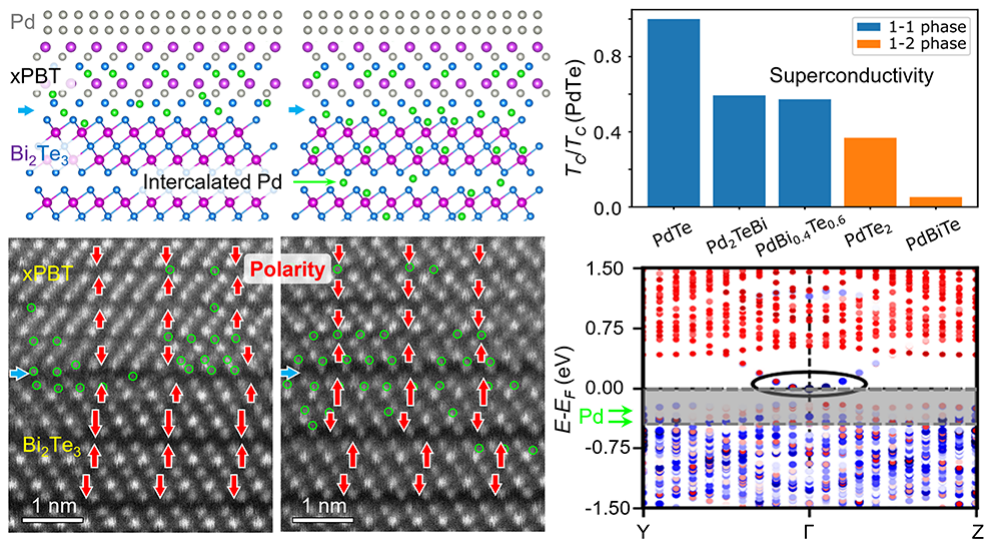

The proximity effect at a highly transparent interface of an *s*-wave superconductor (S) and a topological insulator (TI) provides a promising platform to create Majorana zero modes in artificially designed heterostructures. However, structural and chemical issues pertinent to such interfaces have been poorly explored so far. Here, we report the discovery of Pd diffusion-induced polarization at interfaces between superconductive Pd_1+*x*_(Bi_0.4_Te_0.6_)_2_ (*x*PBT, 0 ≤ *x* ≤ 1) and Pd-intercalated Bi_2_Te_3_ by using atomic-resolution scanning transmission electron microscopy. Our quantitative image analysis reveals that nanoscale lattice strain and QL polarity synergistically suppress and promote Pd diffusion at the normal and parallel interfaces, formed between Te–Pd–Bi triple layers (TLs) and Te–Bi–Te–Bi–Te quintuple layers (QLs), respectively. Further, our first-principles calculations unveil that the superconductivity of the *x*PBT phase and topological nature of the Pd-intercalated Bi_2_Te_3_ phase are robust against the broken inversion symmetry. These findings point out the necessity of considering the coexistence of electric polarization with superconductivity and topology in such S–TI systems.

## Introduction

Majorana zero modes (MZMs) are one of the most exciting research topics in condensed matter systems owing to their potential applications in quantum computation.^1−3^ In essence, MZMs obey non-Abelian braiding statistics.^4^ Under unitary gate operation, the nonlocal encoding of the quasiparticle state makes the computation immune to a certain type of error caused by local perturbation, thus leading to fault-tolerant computation.^5,6^ Relying on nontrivial topological states of matter, the MZMs are predicted to emerge either in spinless *p*-wave topological superconductors (Ss) with one or two dimension^7^ or via proximity-induced superconductivity at interfaces of *s*-wave Ss with topological insulators (TIs).^8^ Meanwhile, possible signatures of MZMs have also been unveiled in a number of systems like semiconductors,^9^ quantum anomalous Hall insulators,^10^ and magnetic atomic chains^11^ such as InSb–NbTiN,^12^ Fe–Pb,^11^ FeTe_0.55_Se_0.45_ superconductors,^13^ EuS–Au,^14^ and LiFeAs.^15^

Given that topological Ss are scarce, implementing the interface-based proximity effect becomes a natural choice to pursue the MZMs and to construct heterostructure-based devices. Among various TIs such as HgTe, BiSb, and PbBi_2_Te_4_,^16−18^ 2D van der Waals (vdW) layered (Bi_1–*x*_Sb_*x*_)_2_Te_3_ has been widely investigated for its tunable topological surface state by chemical doping^19−21^ and control of growth conditions.^22,23^ In spite of the ease in fabricating S–TI nanostructures, e.g., using stencil-lithography-based molecular-beam epitaxy,^24,25^ such hybrid devices usually suffer issues concerning chemical diffusion, electronic structure change, and interfacial dipole layers.^26,27^ Therefore, clarifying the elemental diffusion mechanism and the fundamental physical properties at the interface becomes an urgent task toward creating stable MZMs in such hybrid S–TI devices.

**Figure 1:**
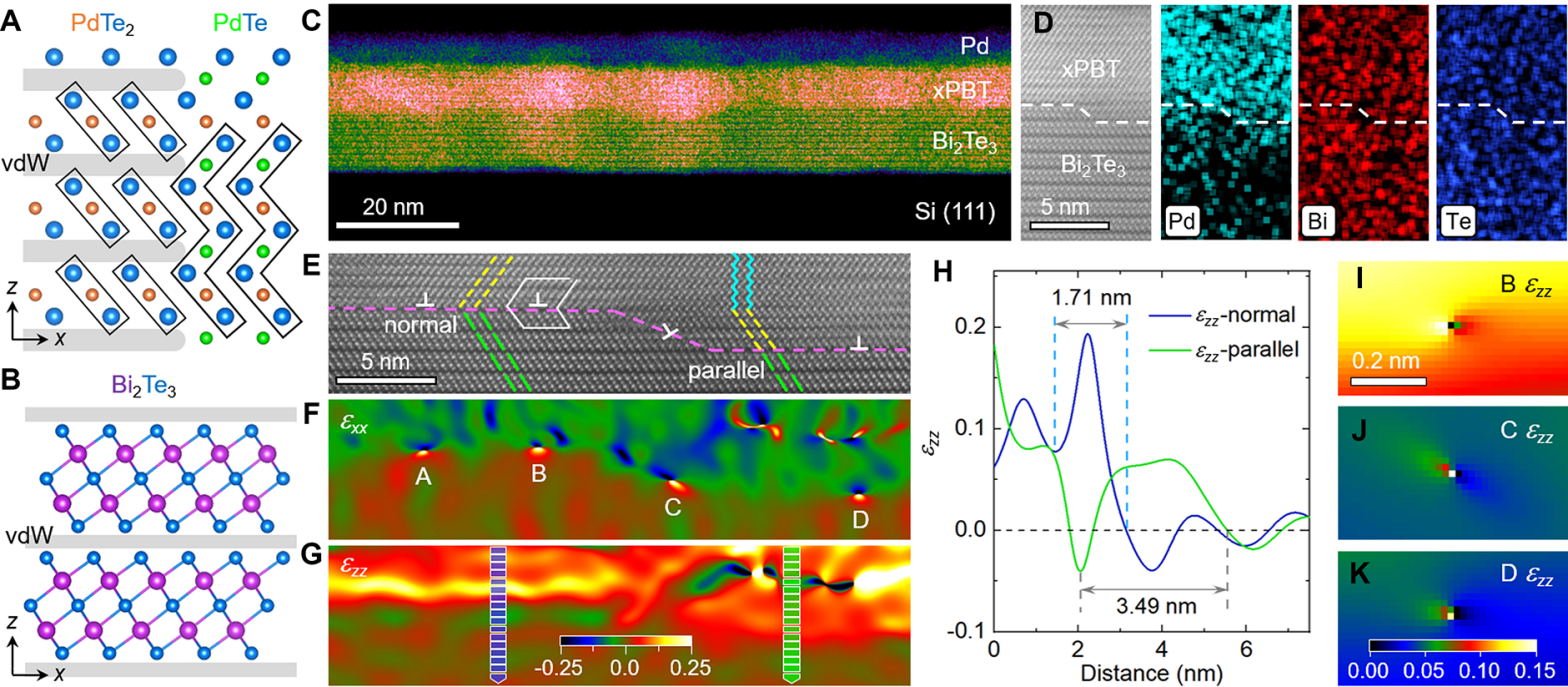
Intermediate *x*PBT phase and interfacial mismatch dislocations. (A, B) Crystal structures of PdTe_2_, PdTe, and Bi_2_Te_3_ viewed along the [100] direction, respectively. The vdW gaps are denoted by gray stripes. (C) Low-magnification HAADF STEM image of a Pd/*x*PBT/Bi_2_Te_3_ heterostructure grown on a Si(111) substrate. (D) HAADF image of a curved interface region and corresponding elemental EDX maps of Pd, Bi, and Te. (E–G) Medium-magnification HAADF image of the interface and GPA strain maps of *ε*_*xx*_ and *ε*_*zz*_, respectively. The Burger vector of the mismatch dislocations is **b⃗** = **a** [100] on the flat (001) planes. The yellow, cyan, and green line segments denote the TLs and QLs, respectively. (H) Local strain line profile of *ε*_*zz*_ extracted from the normal and parallel interfaces illustrated in (G). (I–K) Magnified *ε*_*zz*_ strain maps near dislocations labeled by B, C, and D in (F), respectively.

Lately, PdTe_2_-based Ss have received considerable attention owing to their intriguing band structure and transport property. Studies report that pure PdTe_2_ is a Dirac semimetal with a superconducting *T*_C_ around 1.64 K.^28,29^ By increasing the concentration of Pd, the *T*_C_ of Pd_1+*x*_Te_2_ (*x* ≥ 0) can be increased to 4.5 K in metallic PdTe.^30,31^ Although a potential phase boundary is expected in the structure–composition phase diagram,^31^ a continuous PdTe_2_-to-PdTe solid solution via gradual addition of Pd at the vdW gaps seems to refute the existence of the boundary (Figure 1A). Intriguingly, when metallic Pd is deposited on a TI like Bi_2_Te_3_ (Figure 1B), a PdTe_2_-like superconducting phase (*T*_C_ ≈ 0.6 K) spontaneously forms at the interface through diffusing Pd into the TI.^32^ The newly formed fresh S/TI interface offers an alternative approach to create MZMs via the proximity effect.^33,34^ Other than this, it has been claimed that Pd diffusion into Bi_2_Te_3_ can also lead to formation of a superconductive phase,^35,36^ which indicates controversy about the origin of superconductivity.

In this work, in order to unravel the diffusion-based fundamental physics and device application, the diffusion pathway of Pd into the Bi_2_Te_3_ films grown on Si(111) substrates is investigated by using atomic-resolution scanning transmission electron microscopy (STEM). Unexpectedly, associated with observation of chemical intercalation, the Pd diffusion-induced polarization is observed in the intermediate Pd_1+*x*_(Bi_0.4_Te_0.6_)_2_ (*x*PBT, 0 ≤ *x* ≤ 1) phase and at the *x*PBT/Bi_2_Te_3_ interfaces, i.e., at the normal vs parallel contact interfaces. Apart from disclosing the Pd diffusion pathway, our first-principles calculations reveal robustness of the superconductivity for the *x*PBT and topology for the Pd-intercalated Bi_2_Te_3_ against the broken inversion symmetry. These findings highlight the necessity of exploring polarization–superconductivity–topology coupling in such S–TI systems.

## Results and Discussion

In our experiments, molecular beam epitaxy is used to grow the Bi_2_Te_3_ films (∼18 nm), and a Pd layer (∼6 nm) is deposited on top of Bi_2_Te_3_ to construct the S–TI heterostructures. Distinct from the Nb-capped case,^24^ our high-angle annular-dark field (HAADF) STEM imaging reveals that the capped Pd undergoes a spontaneous diffusion into the Bi_2_Te_3_, leading to formation of an intermediate Pd_1+*x*_(Bi_0.4_Te_0.6_)_2_ (*x*PBT) phase between the Pd surface layer and the Bi_2_Te_3_ film (Figure 1C). Although the Pd penetration depth varies according to the synthetic conditions, e.g., the substrate temperature during metal deposition,^33^ the thickness of the *x*PBT phase is observed to vary in the range of 6.8 to 8.6 nm in this specific case. As for roughness of the *x*PBT/Bi_2_Te_3_ interface, our energy dispersive X-ray spectroscopy (EDS) data reveal that this is attributed to quintuple layer (QL) terraces resulting from varied Pd diffusion depth into the TI (Figure 1D).

The medium-resolution HAADF image shows that the *x*PBT phase is characteristic of a mixture of the PdTe_2_- and PdTe-like triple layers (TLs), which consist of parallel and zigzag-type TLs due to an inhomogeneous distribution of Pd atoms at the vdW gaps. In structure, the PdTe_2_- and PdTe-like phases differ mainly in null and full occupancy of intercalated Pd atoms within the vdW gaps^29^ (Figure 1A). This is substantiated by reproduction of the PdTe phase, previously determined to have the *P*6_3_/*mmc* space group,^30^ via adding one Pd atom at the (0.0, 0.0, 0.5) site in the PdTe_2_ (space group *P*3̅*m*) framework. For clarity, the two structural models are compared and are presented in the Supporting Information (Figure S1). This also supports the absence of the PdTe_2_–PdTe phase boundary in the Pd_1+*x*_Te_2_ (0 ≤ *x* ≤ 1) phase diagram (Figure 1A). One should note that the structure feature of the *x*PBT phase differs drastically from that of superconductive PdBiTe,^37^ which has an intrinsic polar space group *P*2_1_3.

Further, we observe two kinds of interfaces near the QL terraces, a normal interface and a parallel one, which are defined in terms of crystal plane orientation (yellow and green dashed lines) in the TLs and QLs (Figure 1E). In combination with geometric phase analysis (GPA),^38^ we find that the *a*-axis difference between the *x*PBT and Bi_2_Te_3_ phases gives rise to an array of interfacial mismatch dislocations (average spacing ∼7 nm), as manifested by the in-plane *ε*_*xx*_ strain map (Figure 1F). This is similar to mismatch dislocations observed at heterointerfaces of three-dimensional oxides.^39^ Specifically, an out-of-plane *ε*_*zz*_ strain map differentiates the two contact interfaces. At the normal interface, the *c*-axis expansion is highly condensed at the first two TLs (width ∼ 1.7 nm) with *ε*_*zz*-max_ ≈ 0.193 (Figure 1G,H), while at the parallel interface, the lattice expansion extends to about four TLs (width ∼ 3.5 nm) with *ε*_*zz*-max_ ≈ 0.066. By amplifying the *ε*_*zz*_ map, we see further details about the *z*-direction lattice mismatch (Figure 1I–K). Irrelevant to the interfacial contact manner, the local lattices (radius ∼ 0.1 nm) on the left and right side of the dislocation cores are expanded and compressed, respectively. Our image analysis reveals that this arises from asymmetric agglomeration of Pd atoms, manifested by unequal (001) atomic plane numbers near the dislocation cores (see Figure S2 in the Supporting Information).

**Figure 2:**
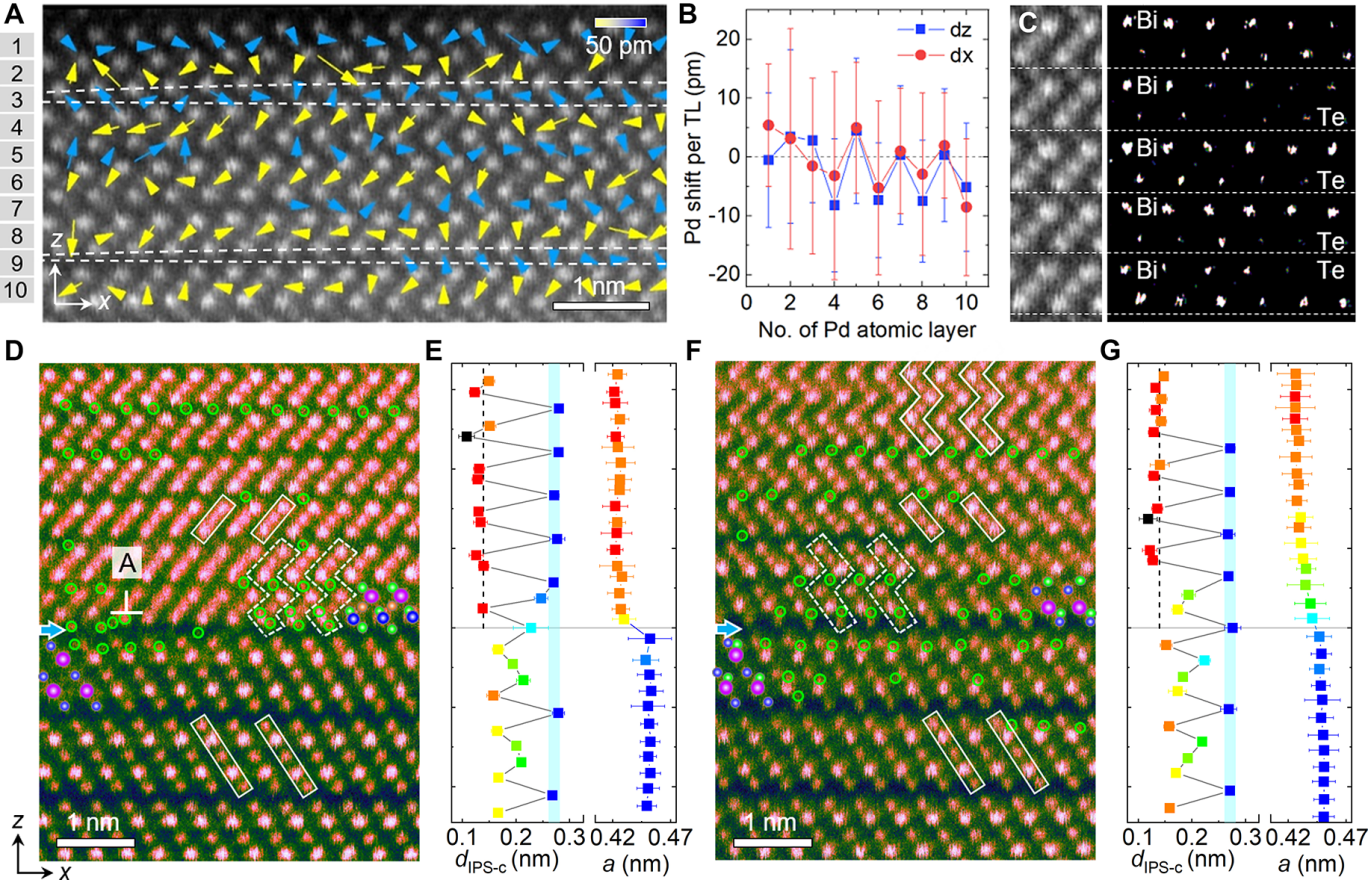
Pd diffusion-induced polarity. (A) HAADF image of the *x*PBT surface with overlapping Pd displacements. (B) Averaged Pd displacements as a function of distance from the surface in the *x*PBT phase. (C) Intensity-filtered HAADF image to illustrate the nanoscale ordering of the Bi and Te arrangement. (D, F) Square-root atomic-resolution HAADF images of [100]-oriented *x*PBT/Bi_2_Te_3_ interfaces with normal and parallel contact, respectively. The discernible Pd columns are annotated by green circles. (E, G) Interplanar spacing (*d*_IPS_) along the *z* and *a* axes measured across the normal and parallel interfaces, respectively. The extreme values of the vdW spacing are determined by experimental (∼0.261 nm)^43^ and theoretical (∼0.281 nm) data reported elsewhere.^44,45^

As for the Pd self-diffusion-induced *x*PBT phase, the HAADF image contrast, proportional to *Z*^1.7^ (*Z*, atomic number),^40^ indicates that the intercalated Pd atoms at the vdW gaps exhibit an irregular occupancy between the TLs (Figure 2A,C). For ease of identification, a HAADF image containing the PdTe_2_- and PdTe-like structures is simulated for comparison (see Figure S3 in the Supporting Information). This leads to nanoscale bending of the TLs (white dashed lines) and possible presence of flexoelectricity, i.e., coupling of the strain gradient with polarization or vice versa. By measuring positions of atomic columns via 2D Gaussian functional fitting,^41^ our mapping reveals a short-range Pd displacement order relative to centers of nearest-neighboring Bi/Te columns (δ_Pd–Bi/Te_) in the TLs (yellow arrows). On the other hand, the Pd atoms at vdW gaps between the TLs tend to exhibit an opposite displacement order (blue arrows). This gives rise to an oscillating polar feature as manifested by line profiles of *δz*_Pd–Bi/Te_ and *δx*_Pd–Bi/Te_, which are averaged along the *x* direction (Figure 2B). Specifically, the nanoscale ordering of Bi and Te atoms along the *z* direction breaks the structural inversion symmetry and thus leads to the emergence of an intrinsic polar order. This is supported by non-negligible charge transfer from Bi/Te to Pd atoms owing to their difference in electronegativity, Bi (χ = 2.02), Te (2.10), and Pd (2.20), as verified in nickel phosphides.^42^

Near the *x*PBT/Bi_2_Te_3_ interface, the HAADF images show that a mixture of parallel and zigzag TLs dominates on the *x*PBT side, which is attributed to fractional occupancy of Pd at the vdW gaps (Figure 2D,F). Nevertheless, more Pd atoms are observed to diffuse into the Bi_2_Te_3_ across the parallel interface, which is evidenced by more Pd atoms located at the interstitial sites within the QLs (green circles). This reveals that the intermediate *x*PBT phase originates from dismembering the Bi–Te bonds of the QLs through the interstitial Pd atoms. On this basis, different dismemberment processes are proposed to understand the Pd diffusion pathways at the two interfaces (see Figure S4 in the Supporting Information). By measuring the interplanar spacing (*d*_IPS_-c), we find that the *a* axis undergoes a sharp transition near the normal interface, while a gradual evolution is observed at the parallel interface (Figure 2E,G). With respect to the average vdW spacing, *d*_0_ = 0.270 ± 0.012 nm, one can see that this value decreases to 0.228 nm and increases to 0.276 nm at the normal and parallel interface, respectively. This unveils that both in-plane and out-of-plane crystal spacings exhibit distinct responses to the suppressed and promoted Pd diffusion at the interfaces.

With consideration of different Pd occupancies at the (0.0, 0.0, 0.5) site of the Pd_*x*_(Bi_0.4_Te_0.6_)_2_ phase, our image simulation study reveals that the experimental specimen thickness is around 43.2 nm and the extracted line profiles indicate that the resolvable Pd concentration is ∼0.35 (Figure S5 in the Supporting Information). This implies that below this critical concentration the intercalated Pd atoms within the vdW gaps and in the QLs of the Bi_2_Te_3_ cannot be directly identified. Associated with structural relaxation, our first-principles calculations on models with different numbers of intercalated Pd atoms further verify that the Pd diffusion at the interstitial positions of the Bi_2_Te_3_ is boosted by high-concentration intercalation of Pd at the vdW gaps (Figures S6 and S7 in the Supporting Information). Specifically, instead of the well-defined zigzag structures, the parallel TLs linked by vdW gaps near the *x*PBT/Bi_2_Te_3_ interface play a crucial role in mediating the Pd diffusion across the interfaces, given that the vdW gaps offer enough space for the dynamic migration of Pd atoms within the lattice matrix (Figure 2D,F).

**Figure 3:**
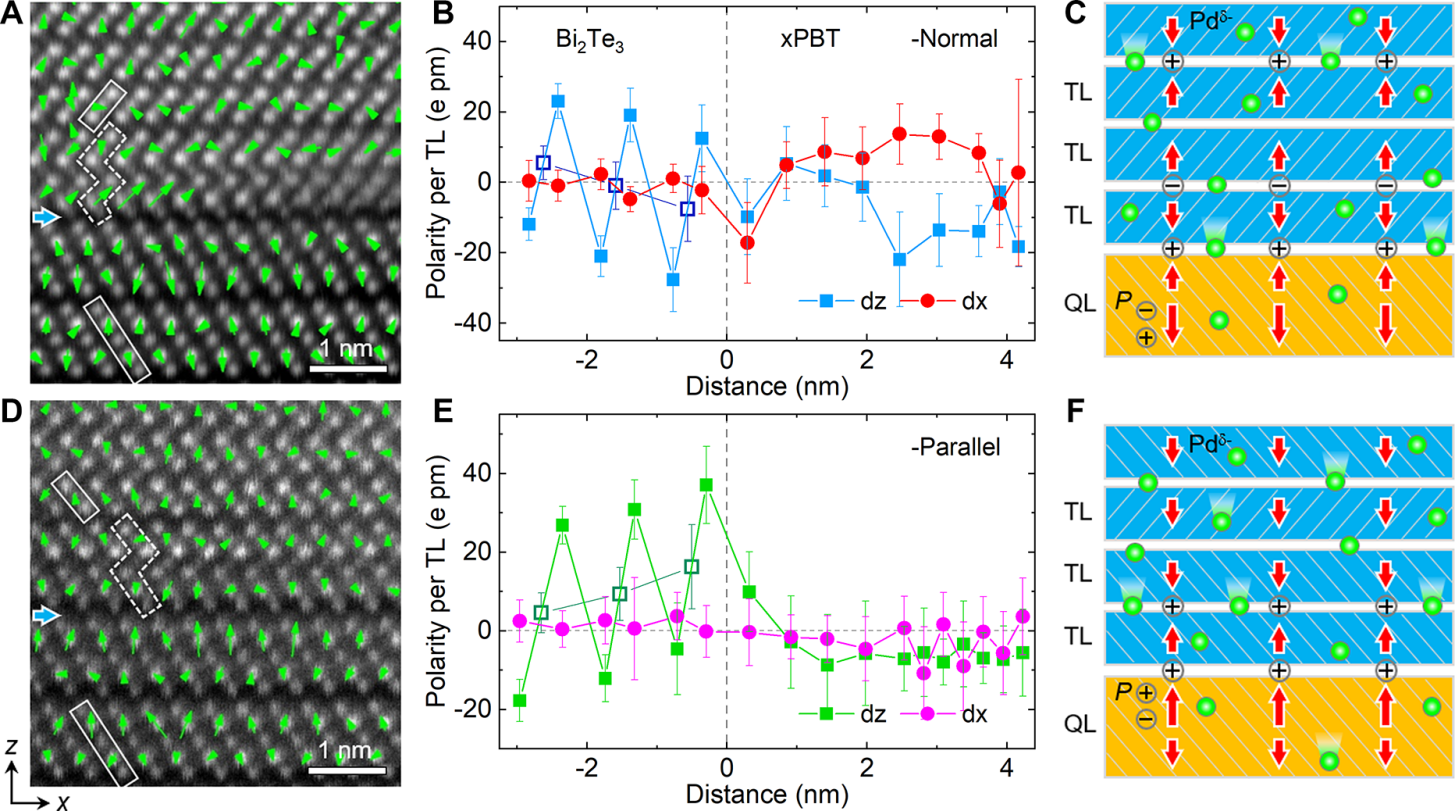
Polarity-mediated Pd diffusion near the interfaces. (A, D) Mapping of polar displacements of Pd and Bi against centers of their nearest-neighboring Te/Bi (δ_Pd–Bi/Te_) and Te columns (δ_Bi–Te_) near the normal and parallel *x*PBT/Bi_2_Te_3_ interface, respectively. (B, E) The polarity profiles (by taking the electron charge equal to 1) per TL near the normal and the parallel interfaces, respectively. The empty squares denote the polarity per QL in the Bi_2_Te_3_. (C, F) Illustration of *z*-axis polarity-mediated Pd diffusion near the head-to-head and tail-to-tail dipole interface, respectively. One should be noted that the electric dipole (pointing from negative to positive charges) direction in the *x*PBT phases (A) and (D) is reversed with respect to the polar displacement shown in (B) and (E), given the larger electronegativity of Pd (denoted by green circles, χ_Pd_ = 2.20) than that of Te (χ_Te_ = 2.10) and Bi (χ_Bi_ = 2.02).

To deeply understand the effect of Pd diffusion, we mapped relative atomic displacements near the two *x*PBT/Bi_2_Te_3_ interfaces (Figure 3A,D). On the *x*PBT side, by averaging the displacement values of δ_Pd–Bi/Te_ along the *x* direction, one can see that the polarity frequently reverses its direction along the *z* direction near the normal interface (Figure 3B). This gives rise to positively charged head-to-head and negatively charged tail-to-tail vdW interfaces, as schematically illustrated in Figure 3C. While near the parallel interface, the polarization gradient and head-to-head configuration result in positively charged wall interfaces (Figure 3E,F). Since Pd is more electronegative and thus more negatively charged than Bi and Te, this indicates that the parallel interface provides a more favorable condition for the diffusion of Pd into the Bi_2_Te_3_ than that at the normal interface. Correspondingly, we see that the in-plane polarity near the normal interface is larger than that near the parallel one, which should be a consequence of local lattice distortion caused by the local Pd concentration (Figure 1I–K).

On the Bi_2_Te_3_ side, we find that the Pd self-diffusion leads to the development of net out-of-plane polarity in the first few QLs near the interface as well (see empty squares in Figure 3B,D). This can refer to the primitive QLs, which are composed of octahedral BiTe_6_ with antiparallel polarity, and the total is null in polarity. Near the normal interface, the overall QL polarity points in the −*z* direction, while near the parallel one, associated with a steady increase of the QL polarity as the interface is approached, the overall QL polarity points to the +*z* direction. This reveals an interface-dependent switching behavior of polarization, which relates to different Pd diffusion pathways. By correlating with the observed Pd distribution, one can see that the +*z*-oriented QL polarity and head-to-head wall interfaces provide an attractive force for the diffusion of Pd atoms into the TI. Given the demand of polarization screening, e.g., at ferroelectric–metal interfaces^41,46^ or at ferroelectric domain walls,^47,48^ these results indicate that instead of electronic screening from the metallic *x*PBT, ionic migration via the Pd diffusion plays a major role in screening the QL polarity in the 2D layered TI. This interprets the suppression and promotion of Pd diffusion at the normal and parallel interface, respectively.

**Figure 4:**
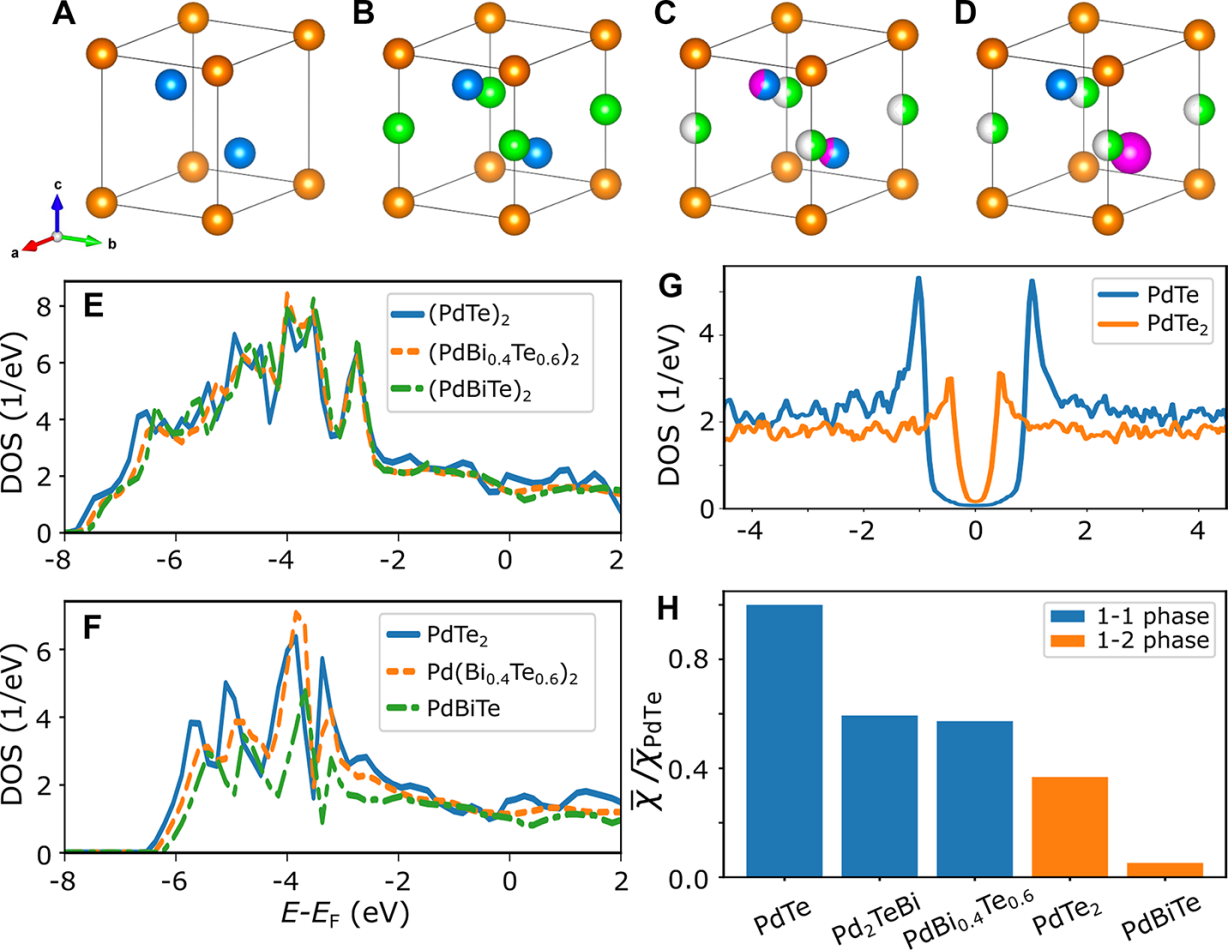
Electronic structure and superconductivity of the *x*PBT phase. (A–D) Crystal structure motifs of (A) PdTe_2_, (B) PdTe, (C) random alloy Pd_*x*_(Bi_0.4_Te_0.6_)_2_, and (D) ordered alloy Pd_1+*x*_(BiTe)_2_ chosen for the DFT calculations. The spheres are color-coded as follows: Pd, orange; Te, blue; Bi, pink; and intercalated Pd, green, where half-filled green spheres refer to either the presence or absence of Pd in the PdTe_*x*_ motif. (E, F) Normal state DOS of (PdBi_0.4_Te_0.6_)_2_ and Pd(Bi_0.4_Te_0.6_)_2_, respectively. (G) Superconducting gap in the DOS of PdTe and PdTe_2_. (H) Magnitude of the average superconducting order parameter of different *x*PBT phases.

To establish a detailed structure–superconductivity relationship, we perform first-principles calculations using the Korringa–Kohn–Rostoker Green (KKR) method^49^ on four structural models of the *x*PBT phases, i.e., pure PdTe and PdTe_2_ phases, a disordered alloy phase of Pd(Bi_0.4_Te_0.6_)_*x*_, and an ordered alloy phase of Pd(BiTe)_*x*_ with *x* = 1 or 2 (Figure 4A–D). According to the BCS theory, the density of states around the Fermi energy, DOS(*E*_F_), exponentially influences the superconducting gap and transition temperature. Compared with the normal-state electronic structures, which exhibit large changes between the PdTe and PdTe_2_ phases, we find that the DOS(*E*_F_) is reduced by about 25–30% as the PdTe and PdTe_2_ phases are disordered by random Bi substitution at their Te sites (Figure 4E,F). Corresponding to an upward shift of the DOS curve, the overall downward shift of *E*_F_ thus indicates that *T*_C_ of the *x*PBT phase is lowered with respect to the PdTe phase.

By taking the similar electron–phonon coupling coefficient (λ) in PdTe and PdTe_2_, with λ = 0.58 and 0.65,^29,50^ our modeling on the intrinsic *s*-wave pairing shows that the superconductivity of the *x*PBT phase is dominated by the electronic degree of freedom and the change in metallicity (see Figure 4G and electron density maps presented in Figure S8 in the Supporting Information). A detailed analysis on atom- and orbital-resolved contributions indicates that the Pd d-electrons are vital to stabilize the superconductivity (see Figure S9 in the Supporting Information). On the one hand, with respect to the PdTe_*x*_ (*x* = 1 or 2) phases (see Table S1 in the Supporting Information),^51−54^ with a ratio of *T*_C_[PdTe_2_]/*T*_C_[PdTe] = 1.7/4.5 = 0.38, its excellent agreement with our calculated ratio of the superconducting order parameter χ, χ[PdTe_2_]/χ[PdTe] = 0.37, unveils that an increasing Bi content tends to reduce the magnitude of χ and thus the *T*_C_ (Figure 4H and Figure S10 in the Supporting Information). One point worth noting is that an ordered Bi–Te arrangement in Pd_2_BiTe (χ = 0.595), introducing an intrinsic polar order in the structure, increases the superconductive *T*_C_ with respect to the disordered Pd(Bi_0.4_Te_0.6_) phase (χ = 0.575), which has less Bi content compared with the Pd(Bi_0.5_Te_0.5_). On the other hand, analogous to the *T*_C_ difference between PdTe and PdTe_2_, a decreasing Pd content also reduces the superconductive *T*_C_ as evidenced in the Pd_2_BiTe and PdBiTe phases. Given that the short-range polar order can be averaged out on the length of tens of nanometers to several micrometers of the superconductive coherence length, we thus speculate that the larger experimental *T*_C_ (compared to pure PdTe_2_) is attributed to mixing of the PdTe- and PdTe_2_-like phases in *x*PBT, which results in an effectively larger average superconducting gap.

**Figure 5:**
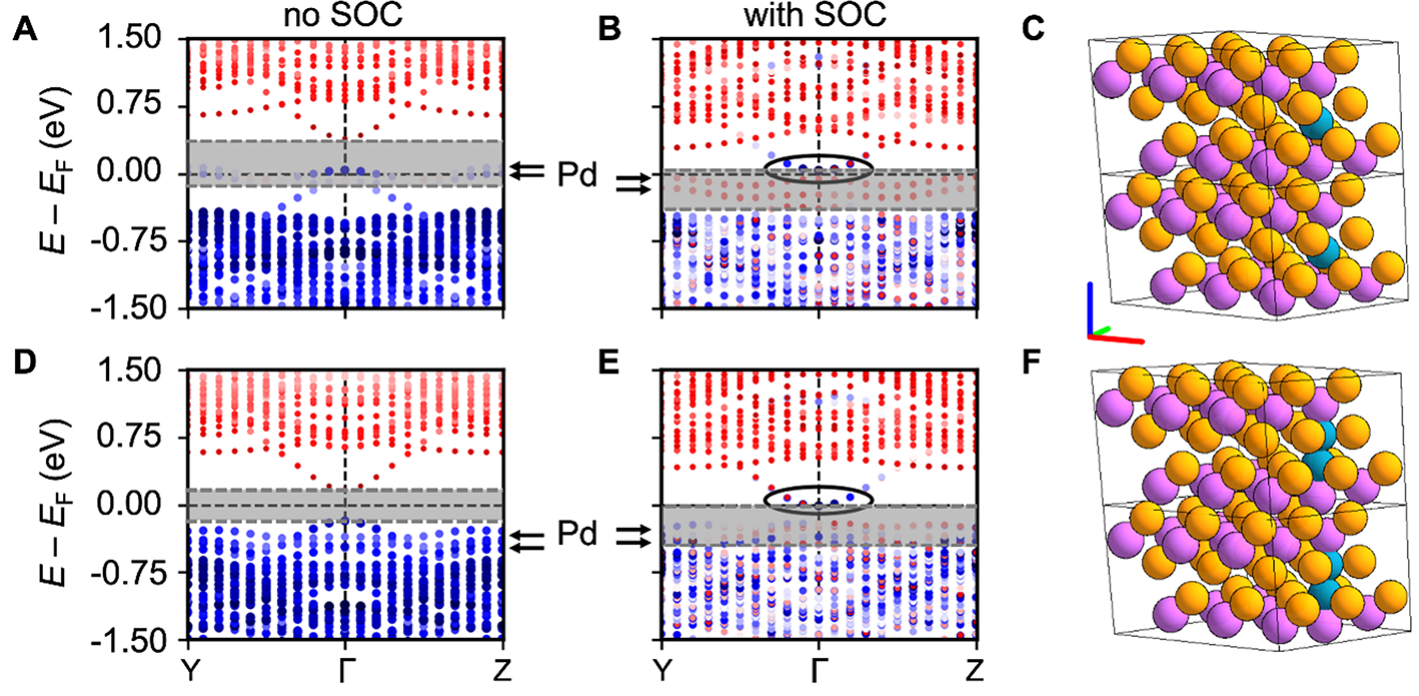
Band inversion in Bi_2_Te_3_ upon Pd diffusion. (A, B) Band structures calculated without and with consideration of the SOC for a 3 × 3 × 1 supercell of Bi_2_Te_3_ (Bi, purple; Te, orange) with a single Pd atom intercalated into the vdW gap shown in (C). (D, E) Band structures without and with SOC for 2 Pd atoms intercalated into the vdW gap shown in (F). The red–white–blue coloring of the bands reflects that the TI conduction band is mainly composed of a Te-p orbital character. The black ellipses highlight the topological band inversion visible in the transferred orbital character from red to blue in the bottom of the conduction band. The gray-shaded areas indicate the TI’s bandgap and location of the flat Pd impurity bands are indicated by the black arrows.

On this basis, we further calculate the band structures of relaxed 3 × 3 × 1 Bi_2_Te_3_ supercells with different concentrations of Pd intercalated into the vdW gaps of the TI (Figure 5A,D and Figure S6 in the Supporting Information). Being consistent with our experimental observation, we find that the high Pd content intercalation mainly leads to structural relaxation along the *z* direction, where the underlying Bi atoms are pushed away from their high-symmetry positions and unequal electric dipoles may form within the QL (see Figure S7 in the Supporting Information). Further, we investigate the robustness of the topological band inversion of the TI upon increasing Pd concentration. It is found that the Pd d-bands form in the bulk bandgap region of Bi_2_Te_3_ (Figure 5B,C). As a function of Pd intercalation concentration, comparison of the band structures with and without consideration of the spin–orbit coupling (SOC) is presented in the Supporting Information (Figure S11). When Pd atoms diffuse into the QL structure at larger Pd concentration, the Pd d-bands move closer to the top of the valence band, and a clear bandgap survives. This reveals that the intercalated Pd atoms and the induced polarity may largely modify the band structure of the Bi_2_Te_3_.

As is known, the topological phase transition in Bi_2_Te_3_ happens when the order of Bi p- and Te p-states becomes inverted around the Γ point due to the SOC.^55^ Following the Te p-character of the bands around Γ upon activating SOC (see Figure S12 in the Supporting Information), our calculation proves that the topological phase transition stays intact at low Pd concentration. Although more Pd-derived impurity bands appear within the TI’s bandgap, the topological band inversion is preserved even at a larger concentration of Pd intercalation into the vdW gap of Bi_2_Te_3_ (Figure 5E,F). One should note that the flat lines around *E*_F_ (highlighted by black arrows in Figure 5) arise from d bands of the intercalated Pd atoms. Although the bands tend to hide the TI’s bandgap, they do not break its topological nature. This is inferred from the surviving band inversion visible in the Te p-character upon including SOC (black ellipses in Figure 5B,E). These results of the bulk electronic structure of Pd-intercalated Bi_2_Te_3_ suggest the robustness of the topological surface states at the *x*PBT/Bi_2_Te_3_ interface. Therefore, a topological superconductor can be expected at such a S/TI heterostructure due to the good proximity effect.^4^

One question worth noting is that if the Pd diffusion happens only at the *x*PBT/Bi_2_Te_3_ interface, will the resulting metallic Pd d-states in the bandgap region stand in the way of realizing a topological superconductor? As is known, the recipe for engineering a topological superconductor in such an S/TI heterostructure requires (i) existence of a topological surface state that can be proximitized; (ii) no other states that are not proximitized and thus close the proximity-induced gap in the electronic structure of the TI. Here we argue that both conditions are met at the *x*PBT/Bi_2_Te_3_ interface. While there are Pd d-derived states in the bandgap of the TI upon Pd diffusion, these are metallic states that are expected to hybridize well with the electronic structure of PdTe_*x*_ due to their compatible orbital character. Because the Pd d-states in the *x*PBT phase are decisive in achieving robust superconductivity, one can conjecture that the Pd-d impurity states may give rise to a sizable proximity gap and will thus be gapped out. The robustness of the topological phase transition upon Pd diffusion further suggests that the topological surface state will be present at the *x*PBT/Bi_2_Te_3_ interface and can be proximitized.

The potential overlap and hybridization with Pd-derived states inside the TI’s bulk bandgap may even be beneficial for the hybridization of the TI’s surface state with the superconductor. This may lead to larger proximity gaps in the topological surface state than those without intercalated Pd atoms. Since the observed dislocations are accompanied by accumulation of Pd atoms on the *x*PBT side, our calculation results (Figure 4H) show that this should rather strengthen the superconductivity of *x*PBT in these regions. Furthermore, since the coherence length, ranging from tens of nanometers to a few micrometers for PdTe and PdTe_2_,^30,50^ is much longer than the dislocation-related structural and compositional fluctuation (less than ∼10 nm), we argue that there will be an averaging out of the superconductive gap on the length scale of the coherence length. Ultimately this leads to a robust superconducting gap in *x*PBT that can proximate the TI surface state for generating topological superconductivity. In addition, no significant charge transfer between the *x*PBT and TI phases is observed in our calculated band structures (Figure S12 in the Supporting Information). This indicates that detrimental band bending effects unveiled in our earlier work on the Nb/Bi_2_Te_3_ interface^56^ are absent, which makes this S–TI interface a good candidate to engineer a topological superconductor.

## Conclusions

In summary, our atomic-scale electron microscopy study reveals two distinct interfaces, the normal and parallel interfaces, between the *x*PBT and Bi_2_Te_3_ phases. On the basis of quantitative image analysis, we find that the inhomogeneous Pd diffusion induces polarization in the *x*PBT phase and the Pd-intercalated Bi_2_Te_3_ phase, respectively. Specifically, it is found that the Pd diffusion is synergistically controlled by interfacial lattice strain and QL polarity, which inherently couple to the diffusion concentration of Pd atoms near the interfaces. Our first-principles calculations point out that the superconductivity of the *x*PBT phase is robust against the inversion symmetry breaking and chemical disorder. Although the Pd diffusion breaks the structural symmetry of the Bi_2_Te_3_, the metallic Pd-derived states in the bulk bandgap do not destroy the topological band inversion. These findings not only unravel the diffusion pathway of metals into the 2D layered TIs, which may apply to Nb-, Cu-, or Sr-doped Bi_2_Se_3_ with nematic superconductivity,^57,58^ but also highlight the necessity of exploring the potential role of electric polarization^59^ on electron pairing when studying MZMs in such S–TI heterostructures.

## Materials and Methods

### Thin Film Growth

The samples were grown as thin films via molecular-beam epitaxy (MBE). First, 10 × 10 mm^2^ Si(111) samples were prepared by a standard set of wafer cleaning steps (RCA-HF) to remove organic contamination and the native oxide. A consecutive HF dip passivates the Si surfaces with hydrogen for the transfer into the MBE chamber (base pressure 5 × 10^–10^ mbar).^60^ To desorb the hydrogen from the surface, the substrates were heated to 700 °C for 10 min and finally cooled to 275 °C. The tellurium shutter was opened several seconds in advance to terminate the silicon surface by Te, which saturates the dangling bonds. Following this, standard Bi and Te effusion cells with vacuum being at 2.2 × 10^–8^ and 5.7 × 10^–7^ mbar were heated to *T*_Bi_ = 460 °C and *T*_Te_ = 260 °C, respectively, for growth of the Bi_2_Te_3_ films. After this, the sample was cooled down to −20 °C (in vacuum), and the Pd was deposited via the e-beam evaporation on top of the Bi_2_Te_3_.

### Scanning Transmission Electron Microscopy Experiments

For electron microscopy observations, cross-sectional lamella specimens with dimensions of around 4 μm × 10 μm were cut along the Si [11̅0] direction using a focused ion beam (FIB, FEI Helios NanoLab 400S) system, and a NanoMill (model 1040) was used to mill down and remove the surface contamination. An FEI Titan 80-200 ChemiSTEM microscope equipped with a HAADF detector and a Super-X energy-dispersive X-ray spectrometer was used to collect the STEM image and EDX results. With a semiconvergent angle at 24.7 mrad, the HAADF images were collected in an angle range of 70–200 mrad. The Dr. Probe software package was used for image simulation,^61^ and CrystalMaker and VESTA software packages were used for drawing the crystal structures. The lattice parameters of the *x*PBT and Bi_2_Te_3_ phases are measured and calibrated by referring to those of the Si substrate. The result shows that the lattice parameters are *a* = 0.425 ± 0.002 nm and *c* = 0.538 ± 0.010 nm for the *x*PBT phase, and *a* = 0.451 ± 0.001 nm and *c* = 3.013 ± 0.015 nm for the Bi_2_Te_3_ phase, respectively.

### First-Principles Calculations

In our all-electron density functional theory (DFT) calculations we use the full-potential relativistic Korringa–Kohn–Rostoker Green (KKR) function method^62^ as implemented in the JuKKR code^63^ as well as the full-potential linearized augmented planewave (FLAPW) code FLEUR.^49^ The FLEUR code is used for structural relaxations, and the KKR method allows us to describe random chemical disorder efficiently via the coherent potential approximation (CPA). The JuKKR code also comes with an extension to the Kohn–Sham–Bogoliubov–de Gennes method, which allows to calculate superconducting properties.^56,64^ The series of DFT calculations in this study are orchestrated with the help of the AiiDA-KKR^65,66^ and AiiDA-FLEUR^67,68^ plugins to the AiiDA infrastructure.^69^ This has the advantage that the full data provenance (including all values of numerical cutoffs and input parameters to the calculation) is automatically stored in compliance with the FAIR principles of open research data.^70^ The complete data set of this project is made publicly available in the materials cloud archive.^71,72^

## Data Availability

The source codes of the AiiDA-KKR plugin,^66^ the AiiDA-FLEUR plugin,^68^ the JuKKR code,^63^ and the FLEUR code^49^ are published as open source software under the MIT license at https://github.com/JuDFTteam/aiida-kkr, https://github.com/JuDFTteam/aiida-fleur, https://iffgit.fz-juelich.de/kkr/jukkr, and https://iffgit.fz-juelich.de/fleur/fleur, respectively. The AiiDA data set containing the DFT calculations of this work is published in the materials cloud archive.^72^ The experimental data are available upon reasonable request.

## Supporting Information Available

Figures of comparison between different structural phases; atomic-resolution *x*PBT/Bi_2_Te_3_ interface; image simulation on the *x*PBT phase; schematic Pd diffusion pathways; HAADF image simulation of Pd intercalation with different occupancy at the vdW gaps; relaxed position of Pd atoms intercalated in the vdW gap of Bi_2_Te_3_; broken symmetry upon Pd interaction in Bi_2_Te_3_; electron density distribution in PdTe and PdTe_2_; atom and orbital resolved anomalous density of PdTe and PdTe_2_; atom resolved anomalous density in different *x*PBT phases; contribution of Pd d orbitals to the band structure of Pd-intercalated Bi_2_Te_3_; contribution of Te p orbitals to the band structure of Pd-intercalated Bi_2_Te_3_; table of experimental superconducting transition temperatures *T*_C_ of different PdTe_*x*_ compounds (PDF)
