# Supplementary material for: Atomic Diffusion-Induced Polarization and Superconductivity in Topological Insulator-Based Heterostructures
Source: ACS Nano. 2023 Dec 21;18(1):571–80. doi: 10.1021/acsnano.3c08601 (PMC10786152; doi:10.1021/acsnano.3c08601)
Supplement: Supplementary file 1 — nn3c08601_si_001.pdf [file nn3c08601_si_001.pdf]

Supplementary Materials

Atomic diffusion-induced polarization and superconductivity in topological insulator-based heterostructures

Xian-Kui Wei[1]*†, Abdur Rehman Jalil[2], Philipp Rüßmann[3,4]*, Yoichi Ando[5], Detlev Grützmacher[2], Stefan Blügel[4], Joachim Mayer[1,6]

[1]Ernst Ruska-Centre for Microscopy and Spectroscopy with Electrons, Forschungszentrum Jülich GmbH, 52425 Jülich, Germany

[2]Peter Grünberg Institute and JARA-FIT, Forschungszentrum Jülich GmbH, 52425 Jülich, Germany

[3]Institute for Theoretical Physics and Astrophysics, University of Würzburg, 97074 Würzburg, Germany

[4]Peter Grünberg Institute and Institute for Advanced Simulation, Forschungszentrum Jülich GmbH and JARA, 52425 Jülich, Germany

[5]Physics Institute II, University of Cologne, Zülpicher Str. 77, 50937 Köln, Germany

[6]Central Facility for Electron Microscopy, RWTH Aachen University, Ahornstraße 55, 52074 Aachen, Germany

*Corresponding author Emails: xkwei@xmu.edu.cn; p.ruessmann@fz-juelich.de

†Present address: College of Chemistry and Chemical Engineering, Xiamen University, 361005, Xiamen, China

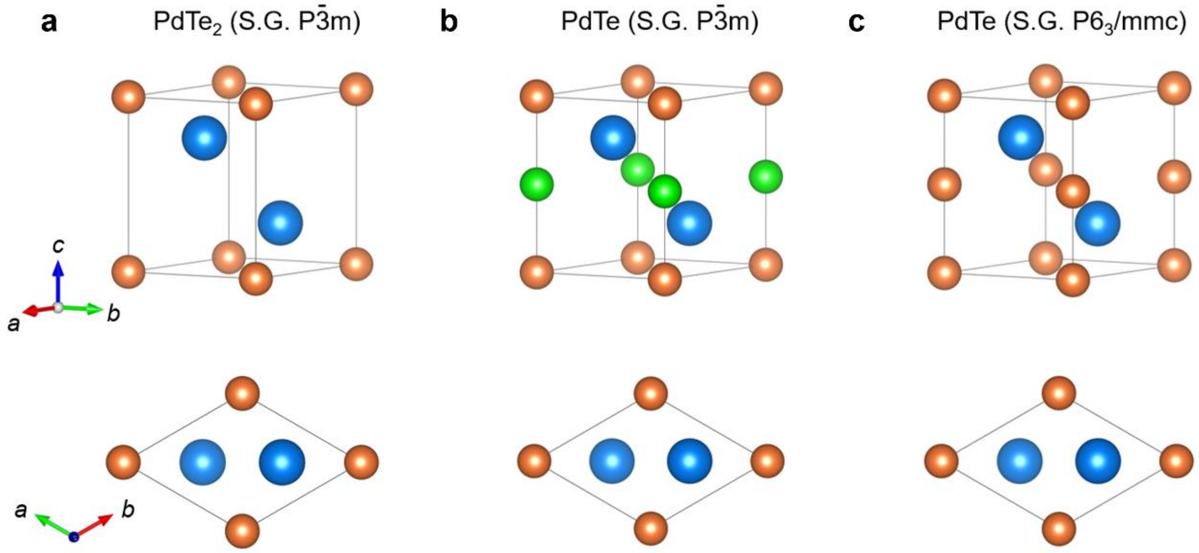

Figure S1. Comparison between different structural phases. a-c) Crystal structures of $PdTe_2$ and PdTe with space group (S.G.) $P\bar{3}m$, and PdTe with S.G. $P6_3/mmc$, respectively. The atom types are colored in blue (Te), orange (Pd) and green (intercalated Pd). The top row is the stereo view and the bottom row is the projection view along the *c* axis.

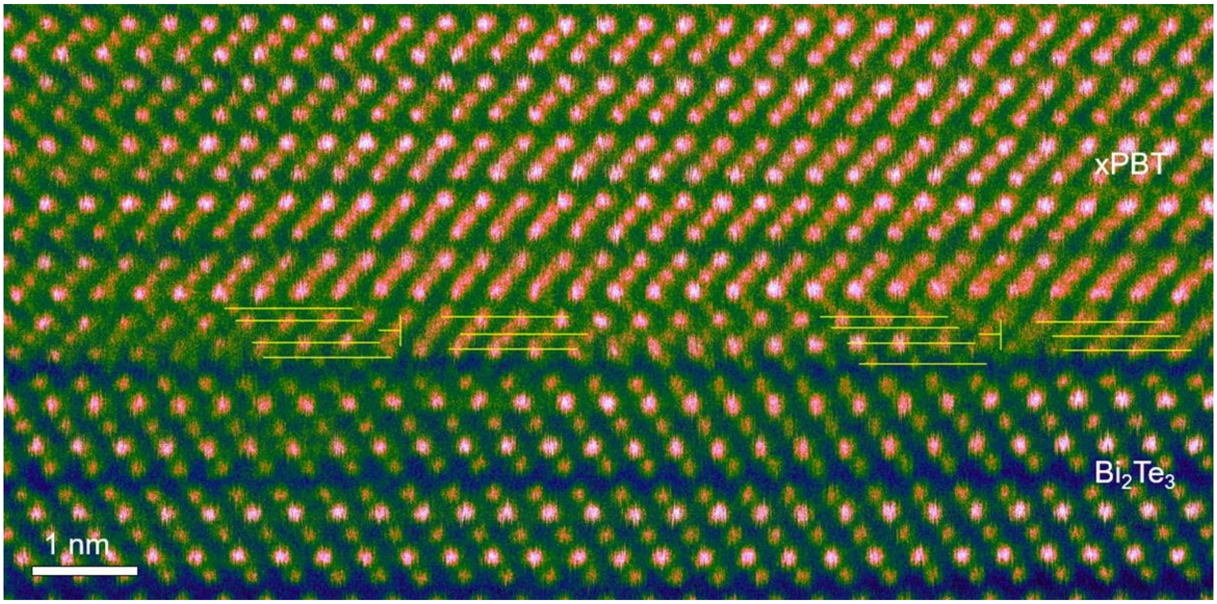

Figure S2. Atomic-resolution xPBT/Bi_2Te_3 interface. Yellow solid lines are marked on the HAADF image to show an additional component of the interfacial mismatch dislocation caused by asymmetric Pd accumulation.

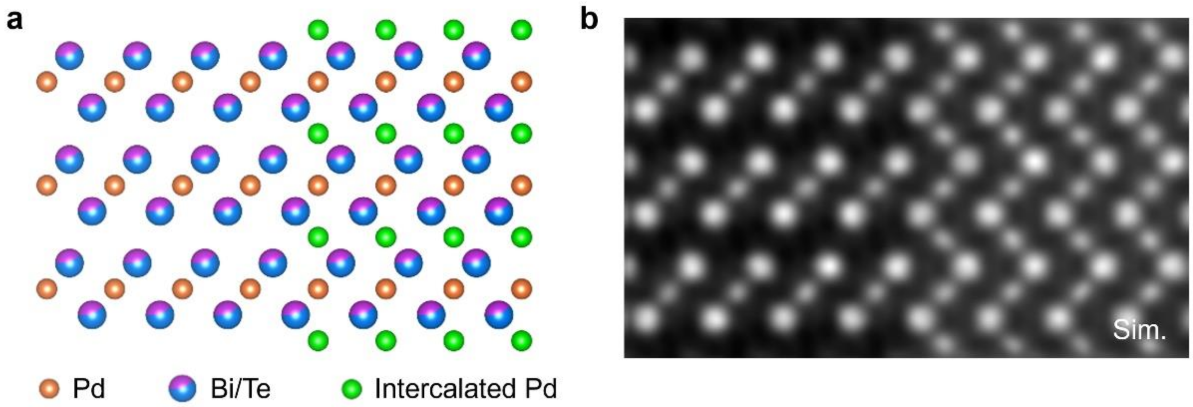

Figure S3. Image simulation on the xPBT phase. a) Structural model with and without Pd intercalation. **b**) Simulated HAADF image with a thickness of 43.2 nm.

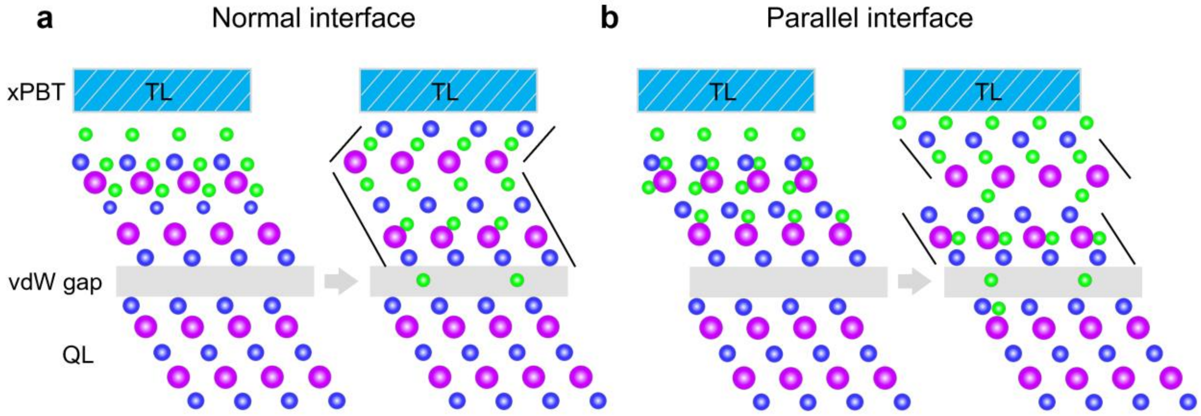

Figure S4. Schematic Pd diffusion pathways. a,b) Possible formation of normal-mode and parallel-mode contact interfaces, respectively. Although the breaking of Bi-Te bonds by intercalated Pd atoms dominates in both scenarios, it seems that the intercalation at the vdW gaps may suppress the chemical diffusion, as manifested by the highly condensed ε_{yy} strain.

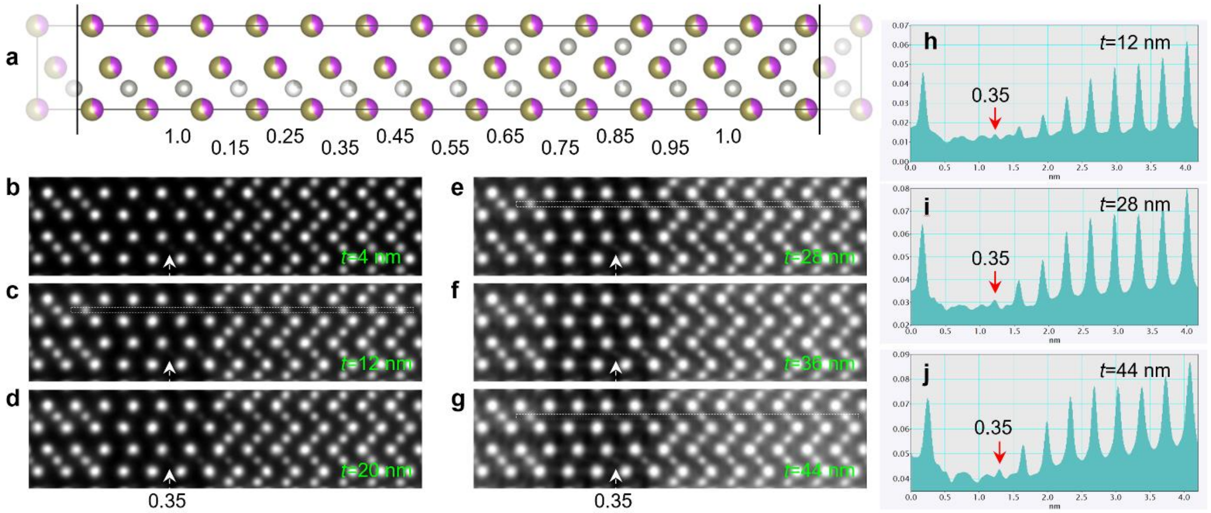

Figure S5. HAADF image simulation of Pd intercalation with different occupancy at the vdW gaps. a) Structure model. **b-g**) Simulated HAADF images with thickness ranging from 4 to 44 nm. **h-j**) Intensity profiles extracted from images of c), e) and g), respectively.

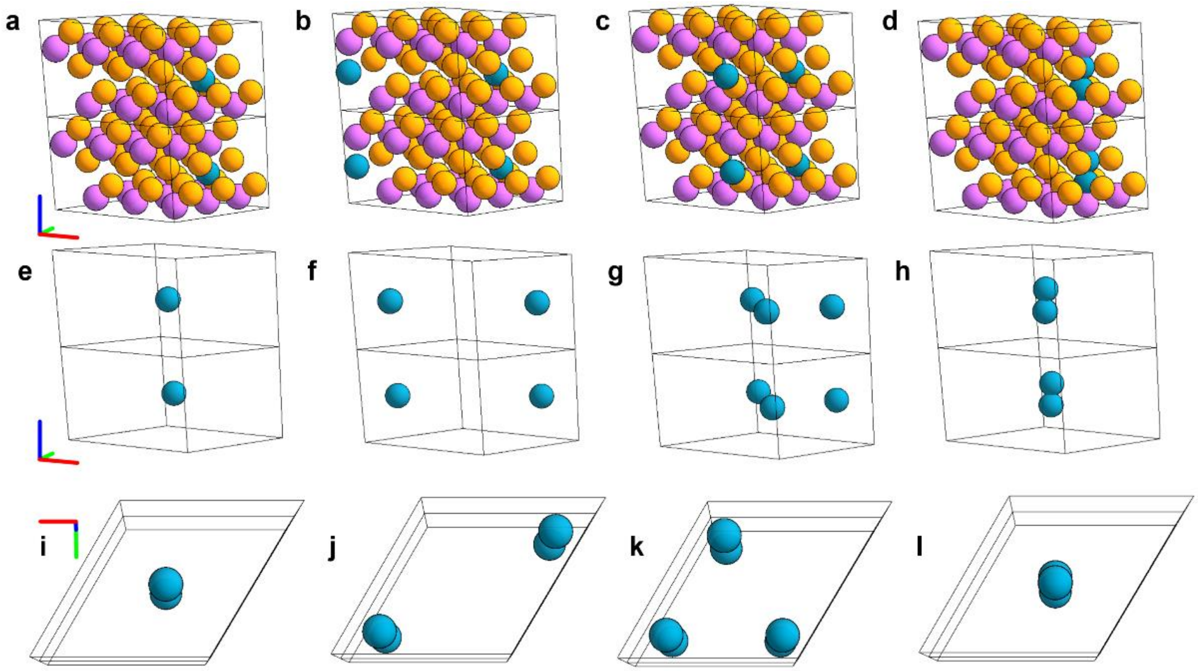

Figure S6. Relaxed position of Pd atoms intercalated in the vdW gap of Bi_2Te_3. a-d) Relaxed positions of one to three Pd atoms in a $3 \times 3 \times 1$ Bi_2Te_3 supercell, calculated with the FLEUR code. The atom types are Pd (blue), Bi (pink) and Te (orange). **e-h**) Side view of only the Pd atoms, respectively. **i-l**) Top view of the Pd atoms in the unit cells, respectively. When the Pd concentration is low, a-c), the individual Pd atoms remain in the center of the vdW gap. Only when two Pd atoms occupy the same hollow site d), the Pd atoms repel each other and are pushed deeper into the QL, ending up at roughly the same height as the neighboring Te atoms.

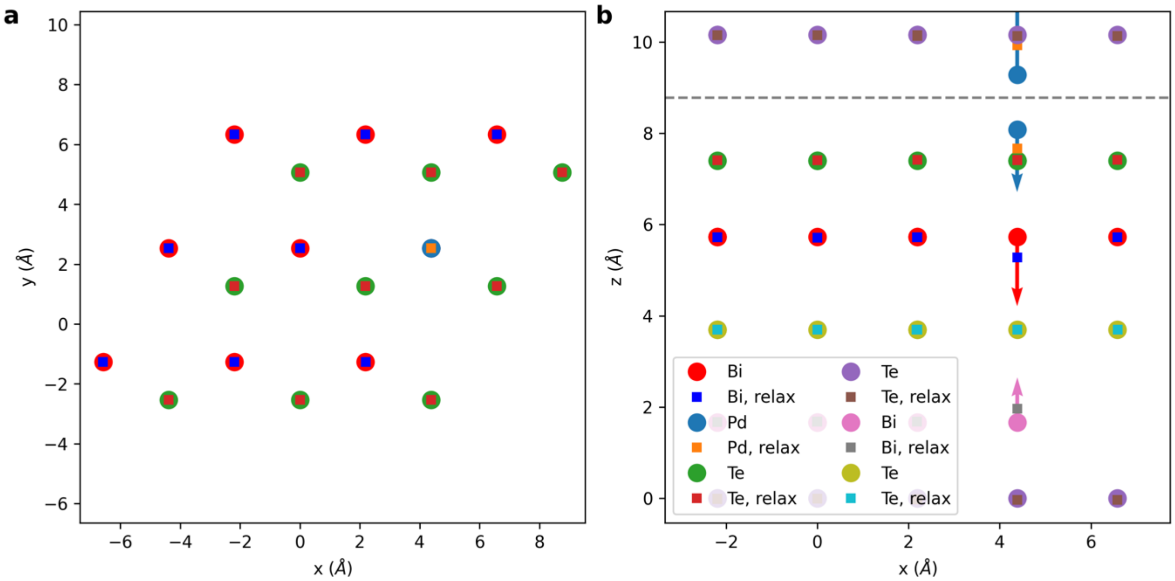

Figure S7. Broken symmetry upon Pd interaction in Bi_2Te_3. a) [001] view of the Pd-intercalated Bi_2Te_3. **b)** [$1\bar{1}0$] view showing the relaxation of Bi_2Te_3 with two Pd atoms intercalated at the vdW (grey dashed line) gap as illustrated in Fig. S6d. The original and relaxed positions are indicated by colored spheres and squares, respectively. The direction of the atom relaxation is highlighted by color arrows. One can see that the atoms relax only considerably along *z*-direction of Bi_2Te_3. With inward movement of Pd atoms into the outermost Te layer of the Bi_2Te_3 quintuple layer (QL), the underlying Bi atoms (red circle with arrow and relaxed blue square) are attracted towards the center of the QL.

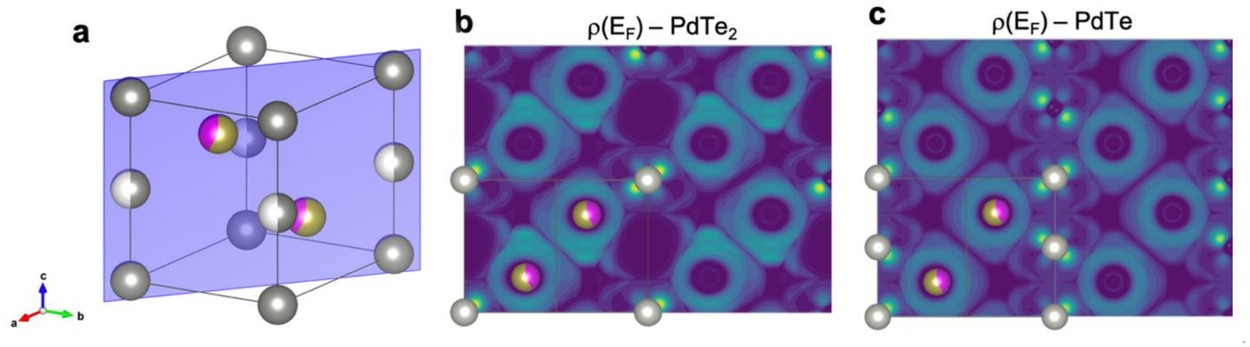

Figure S8. Electron density distribution in PdTe and $PdTe_2$. a) Unit cell of $PdTe_x$ where the blue shaded surface highlights the plane in which the charge density at the Fermi level is visualized for **b)** $PdTe_2$ and **c)** PdTe. The additional Pd atoms in c) add additional states (zig-zag pattern in vertical direction along the line formed by Pd atoms) that leads to an increased metallicity and enhanced superconductivity in PdTe compared to $PdTe_2$.

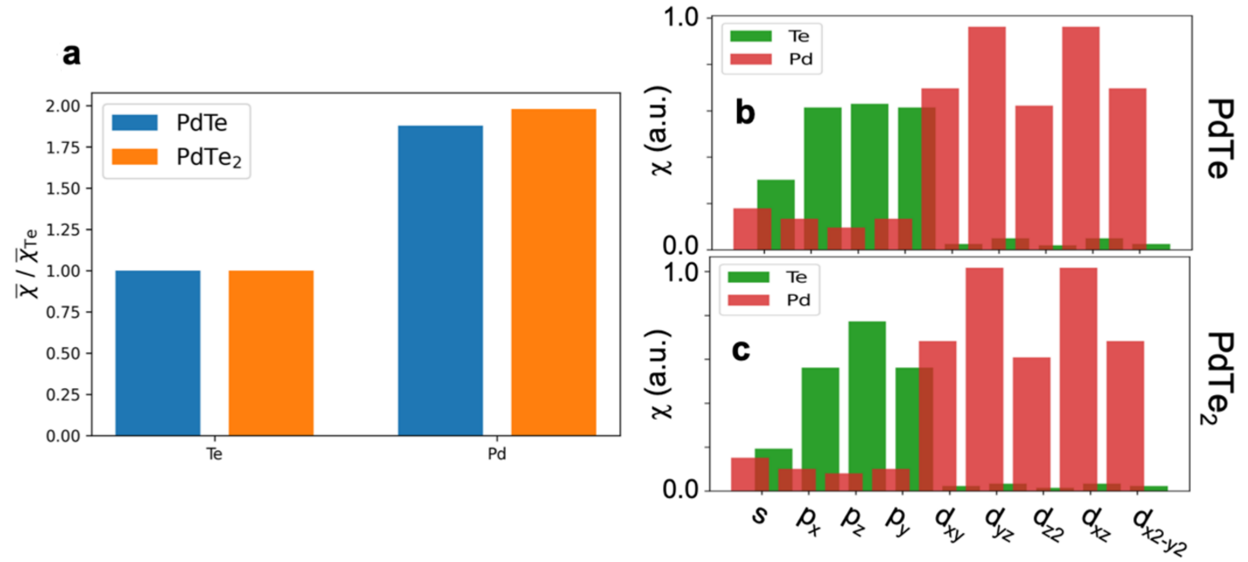

Figure S9. Atom and orbital resolved anomalous density of PdTe and $PdTe_2$. a) Atom resolved anomalous density for PdTe (blue) and $PdTe_2$ (orange) relative to the contribution of one Te atom. **b,c)** Atom and orbital resolved anomalous density for PdTe (b) and PdTe2 (c).

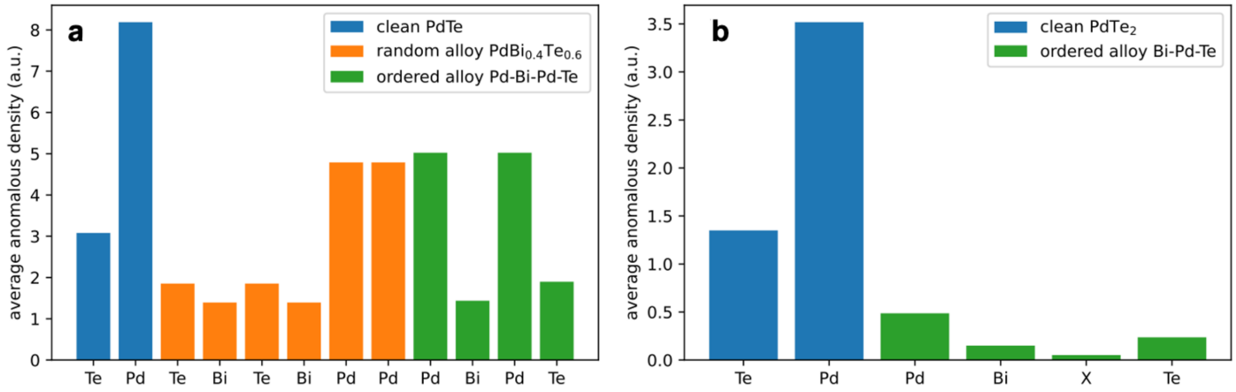

Figure S10. Atom resolved anomalous density in different xPBT phases. a) Atom-resolved anomalous density in clean PdTe (blue), the random alloy $PdBi_{0.4}Te_{0.6}$ and in the ordered alloy Pd_2BiTe. **b)** Atom resolved anomalous density in clean $PdTe_2$ and the ordered alloy phase of PdBiTe.

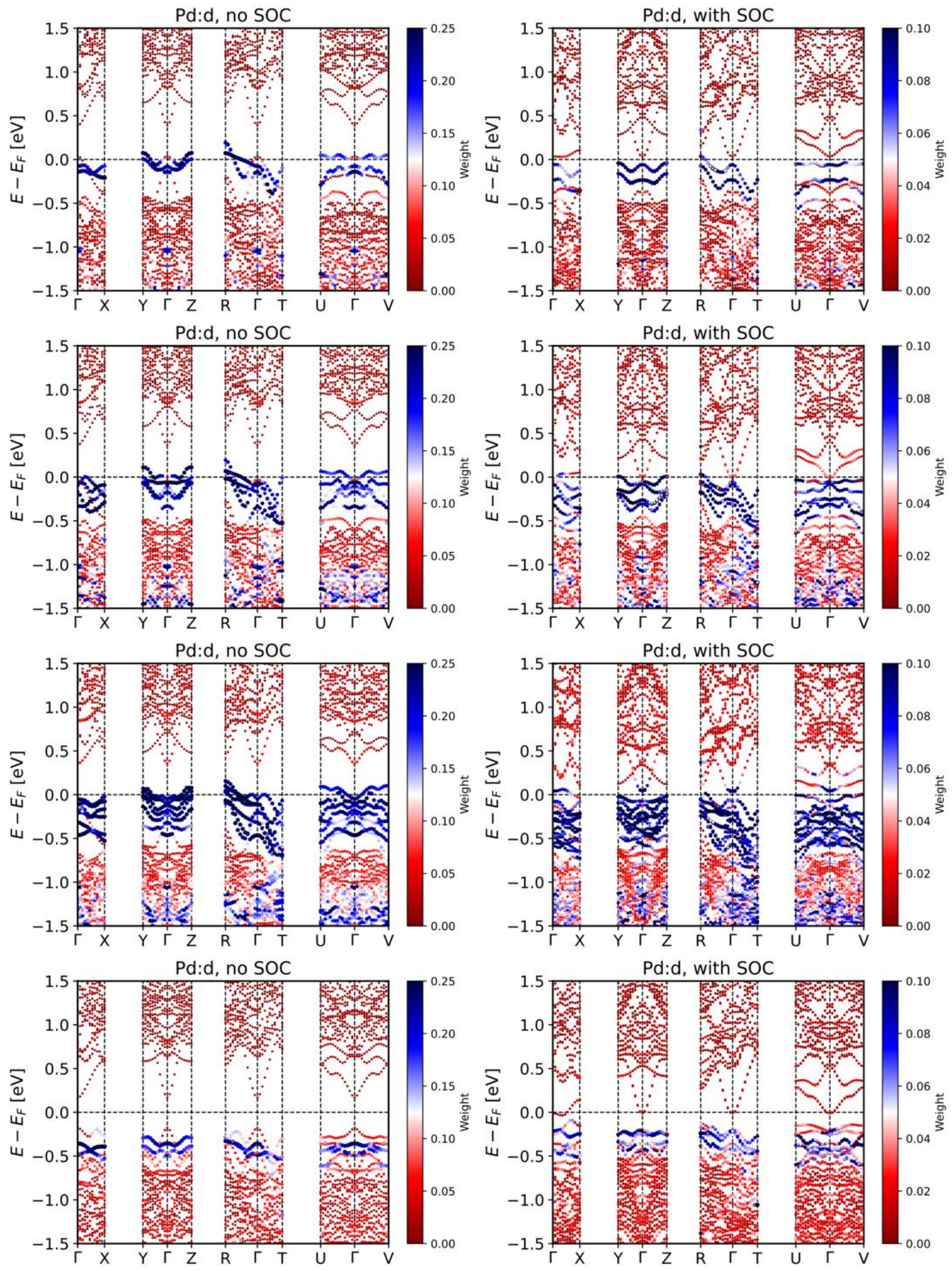

Figure S11. Contribution of Pd *d* orbitals to the band structure of Pd-intercalated Bi_2Te_3. The four rows correspond (from top to bottom) to the structural models given in Fig. S6 (a-d) for one to three Pd atoms intercalated into the vdW gap of a $3 \times 3 \times 1$ Bi_2Te_3 supercell. The last row corresponds to the experimentally observed position of Pd atoms at larger concentration. The Pd *d*-states are highlighted in blue. Left column: band structure without SOC, right column: band structure including SOC.

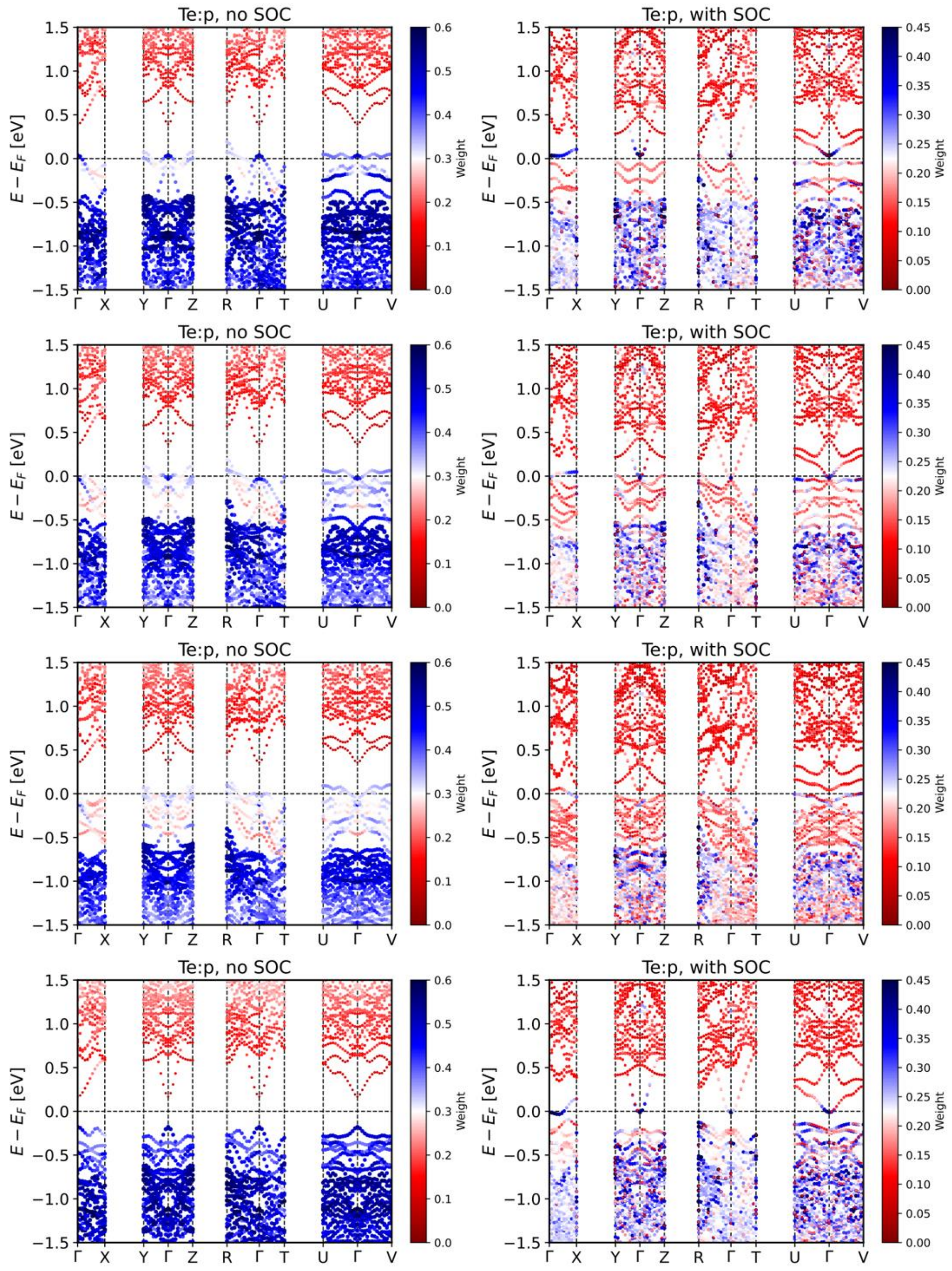

Figure S12. Contribution of Te *p* orbitals to the band structure of Pd-intercalated Bi_2Te_3. Layout of the plots as in Figure S8 but the blue colors highlight the Te *p*-states for the four structural model given in Fig. S6(a-d). Without SOC (left column) the valence band is made up of predominantly Te *p*-states whereas the conduction band (CB) is of predominantly Bi *p* character. With SOC (right column) the topological band inversion is visible around Γ as the blue color at the bottom of the CB. This is only weakened in the third row where many Pd *d*-states are present inside the Bi_2Te_3 in the band gap (cf. Fig. S10).

Table S1. Experimental superconducting transition temperatures T_C of different $PdTe_x$ compounds.

**Material**	**T_C [K]**	**References**
PdTe	4.5	Refs. 30, 31
$PdTe_2$	~1.7	Refs. 28, 29, 50, 51
$Pd(Te,Se)_2$	2.74	Ref. 52
Cu-intercalated $PdTe_2$	2.4	Ref. 49
Pd-Bi_2Te_3	0.67-1.22	Ref. 33
